# Soluble Urokinase-type Plasminogen Activator Receptor (suPAR) as a Biomarker of Neurodysfunction

**DOI:** 10.1007/s12017-026-08912-1

**Published:** 2026-03-12

**Authors:** Victoria Linden de Rezende, Khiany Mathias, Lucineia Gainski Danielski, Tatiana Barichello, Fabricia Petronilho

**Affiliations:** 1 Laboratory of Experimental Neurology, Graduate Program in Health Sciences, University of Southern Santa Catarina (UNESC), Criciúma, SC Brazil; 2 Department of Surgery, Division of Surgical Sciences, University of Texas Medical Branch, Galveston, TX USA; 3 Faillace Department of Psychiatry and Behavioral Sciences, Translational Psychiatry Program, McGovern Medical School, The University of Texas Health Science Center at Houston (UTHealth), Houston, TX USA

**Keywords:** SuPAR, Systemic inflammation, Biomarker, BBB damage, CNS disorders

## Abstract

The inflammatory response is essential for host defense, but its persistence can lead to chronic systemic inflammation (CSI). Soluble urokinase-type plasminogen activator receptor (suPAR) has emerged as a reliable biomarker of CSI because elevated levels consistently indicate the presence and progression of chronic disease as well as increased mortality risk. There is growing evidence that CSI influences neurovascular regulation, including changes in blood-brain barrier (BBB) integrity, which suggests that suPAR may also be relevant to central nervous system (CNS) processes. This narrative review summarizes current findings on suPAR in CSI and examines its emerging implications for CNS. Higher suPAR concentrations have been linked to working memory impairment, executive dysfunction and worse clinical outcomes after brain injury. Evidence also indicates that suPAR reflects neuroinflammatory activity and BBB disruption, especially in conditions marked by heightened immune activation. However, available studies differ widely in design, sample type, follow-up duration and population characteristics, which limits mechanistic interpretation. Although suPAR appears to be a promising biomarker connecting systemic inflammation to CNS dysfunction, its role within the brain remains unclear. Future studies should determine its cellular origin, clarify its involvement in inflammatory signaling pathways and establish its predictive and prognostic value.

## Introduction

The inflammatory response is a fundamental defense mechanism that typically remains localized to the site of injury, acting to eliminate pathogens, limit infection, and facilitate tissue repair. When the acute immune response fails to fully resolve the harmful stimulus, the immune system can become persistently activated, leading to a state of chronic inflammation (Rasmussen et al., 2021). It is important to note that acute inflammation has a rapid onset and becomes severe in a short period, with symptoms typically lasting only a few days. In contrast, chronic inflammation, also referred to as long-term inflammation, persists for prolonged periods, ranging from months to years (Zotova et al., 2023). It is this chronic form, more specifically in its systemic presentation, that will be addressed in this manuscript.

In recent years, soluble urokinase-type plasminogen activator receptor (suPAR) has emerged as a validated and promising biomarker of chronic systemic inflammation (CSI), as elevated concentrations reliably indicate the presence and progression of chronic diseases as well as an increased risk of early mortality (Artusa et al., 2025; Rasmussen et al., 2017; Sehgal et al., 2024). Growing evidence further indicates that CSI can strongly influence neurovascular regulation, particularly by disrupting the permeability and structural integrity of the blood-brain barrier (BBB) (Galea, 2021). Considering this dynamic bidirectional communication between the periphery and the central nervous system (CNS), it is reasonable to propose that biomarkers such as suPAR may also play a significant role in CNS-related processes and brain function.

However, the literature regarding the mechanisms through which suPAR acts within the CNS remains limited. Several studies have correlated elevated levels of this protein with working memory deficits (De Almeida et al., 2022; Gianella et al., 2019), executive function decline (Shell et al., 2025), and worse outcomes following brain injury (Onatsu et al., 2017; Śmiłowska et al., 2022; Sajanti et al., 2025). suPAR is primarily discussed as a biomarker of CSI associated with CNS dysfunction. While this molecule is known to amplify inflammatory signaling pathways in the periphery (Rasmussen et al., 2021; Thunø et al., 2009; Wang et al., 2025), current evidence in CNS-related conditions remains largely associative, and a direct effector role within the brain cannot yet be established. Accordingly, this short review aims to examine and integrate current evidence on suPAR as a biomarker of CSI and to explore its potential implications within the CNS.

## SuPAR Biology

The extracellular matrix (ECM) is a complex network of proteins and polysaccharides that is secreted, assembled, and shaped by cells, providing physical support and structural organization to tissues (Hynes, 2009; Kular et al., 2014). In addition, it promotes cell communication, influencing processes such as proliferation, differentiation, migration, and survival (Saraswathibhatla et al., 2023; Smith & Marshall, 2010). In this context, the urokinase-type plasminogen activator receptor (uPAR) is a molecule composed of three domains (D1, D2, and D3), located on the cell surface and anchored by glycosylphosphatidylinositol (GPI). This receptor plays an essential role in the degradation and remodeling of the ECM through extracellular proteolysis (Smith & Marshall, 2010).

**Fig. 1:**
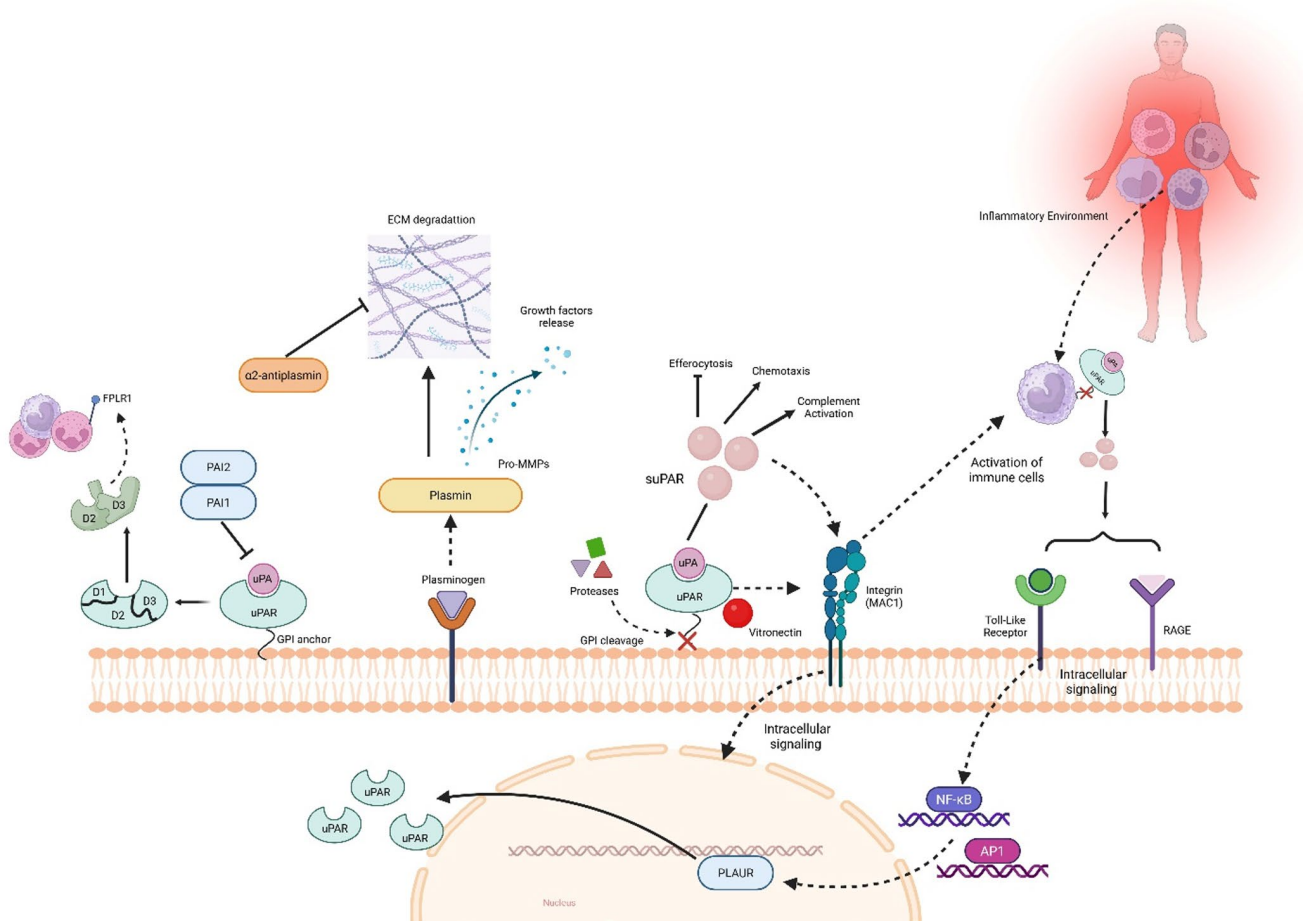
Schematic representation of the uPA-uPAR system and the generation of the soluble receptor suPAR in the context of chronic systemic inflammation (CSI). uPAR, anchored to the membrane by a GPI moiety, interacts with uPA and regulates the conversion of plasminogen into plasmin, promoting extracellular matrix (ECM) degradation, activation of pro-MMPs, and release of growth factors, processes that are controlled by inhibitors such as PAI-1, PAI-2, and α2-antiplasmin. Cleavage of uPAR generates suPAR, which can be further fragmented into the D1 and D2-D3 domains, retaining biological activity, including chemotactic effects and amplification of inflammatory signaling. suPAR can stimulate immune cell activation and migration through interactions with integrins, such as MAC-1, and these immune cells may also act through binding to membrane receptors, including Toll-like receptors (TLR) and the receptor for advanced glycation end products (RAGE), triggering intracellular pathways associated with the inflammatory response. Under conditions of persistent immune activation, immune cells release suPAR into the circulation, reflecting the intensity and chronicity of the systemic inflammatory response

uPAR exerts its function mainly through the regulation of the plasminogen activation system. It binds to both the active urokinase enzyme (uPA) and its inactive form (pro-uPA). The interaction between uPA and uPAR promotes the conversion of plasminogen into plasmin, a protease responsible for degrading ECM components such as collagen and fibronectin (Blasi & Carmeliet, 2002; Smith & Marshall, 2010). Moreover, plasmin activates pro-matrix metalloproteinases (pro-MMPs), which also participate in the degradation process. As a result, growth factors previously sequestered within the matrix are released, promoting tissue regeneration and cell growth. This pathway is tightly regulated by inhibitors such as PAI-1, PAI-2, and α2-antiplasmin, which prevent excessive ECM degradation (Ye, 2001). Additionally, uPAR interacts with other proteins, including integrins and vitronectin, contributing to cell adhesion and signaling (Smith & Marshall, 2010) (Fig. 1).

As mentioned, uPAR is anchored to the cell membrane through a GPI linkage; however, this anchor can be cleaved, generating a soluble form known as suPAR (soluble uPAR) (Blasi & Carmeliet, 2002; Ploug & Ellis, 1994). Both uPAR and suPAR can undergo further cleavage in the region connecting domains D1 and D2, resulting in two fragments: D1, which contains the uPA-binding site, and D2D3, which exhibits chemotactic activity (Ploug & Ellis, 1994). Thus, even after being released from the cell surface, suPAR retains important biological functions, now acting in a circulating and distant manner, signaling to other cells and serving as a mediator or marker of proteolytic and inflammatory tissue activity (Smith & Marshall, 2010).

Reiser et al. (2026) demonstrated in their study that dysregulation of innate immunity, induced by conditions such as diabetes, hypertension, infections, and smoking, increases circulating suPAR levels and/or promotes the generation of its D2-D3 fragments, which exert organ-specific effects. While intact suPAR primarily contributes to systemic inflammatory activation and endothelial dysfunction, the D2-D3 fragments interact with integrins, triggering renal podocyte injury, pancreatic β-cell dysfunction, and progression of atherosclerosis. Interestingly, beyond biological and environmental factors influencing suPAR levels, sex-specific differences have been reported, with women generally exhibiting higher baseline suPAR concentrations than men; however, the prognostic value of suPAR appears comparable between sexes when sex-adjusted thresholds are applied (Haupt et al., 2014; Mehta et al., 2020).

Under physiological conditions, suPAR contributes to tissue remodeling and repair; however, when present at elevated levels, it may be associated with pathological processes such as CSI, tumor invasion, and, more recently, neurodegenerative diseases (Bahrami et al., 2025; De Almeida et al., 2022; Jo et al., 2009; Pietras, 2017; Sajanti et al., 2025; Shell et al., 2025). It is important to note that several therapeutic approaches have been proposed to reduce circulating suPAR levels, including WAL0921, a first-in-class anti-suPAR monoclonal antibody developed by Walden Biosciences, which is currently being evaluated in phase II clinical trials in individuals with glomerular kidney diseases (ClinicalTrials.gov NCT06466135).

## SuPAR and Systemic Inflammation: A Bridge To the Brain

The inflammatory process is an essential defense mechanism of the body, generally restricted to the site of injury, with the purpose of eliminating pathogens, containing infections, and promoting tissue repair. When the acute immune response is not sufficient to eliminate the aggressor agent, a persistent activation of the immune system occurs, characterizing a chronic inflammatory state (Rasmussen et al., 2021). Unlike these localized responses, inflammation may also acquire a chronic and systemic nature, affecting multiple physiological systems and contributing to the development of several diseases, ultimately increasing morbidity and mortality (Furman et al., 2019).

Recently, suPAR has been validated and highlighted as a promising biomarker for CSI. During such conditions, uPAR is cleaved from the surface of activated immune cells, leading to an increase in its soluble form, suPAR. Interestingly, elevated levels of this protein allow the detection of the presence, progression, and premature mortality associated with chronic diseases (Artusa et al., 2025; Hayek et al., 2020; Rasmussen et al., 2017; Sehgal et al., 2024). Traditionally, acute-phase markers such as C-reactive protein (CRP), fibrinogen, and erythrocyte sedimentation rate (ESR) are used to assess acute and transient inflammatory processes (Rasmussen et al., 2021). In contrast, suPAR stands out for more accurately reflecting chronic, low-grade immune activation, remaining elevated even in the absence of acute inflammation.

Accumulating evidence has identified suPAR as an important marker in cardiovascular and renal diseases, sepsis, and other inflammatory disorders (Desmedt et al., 2017; Hodges et al., 2015; Rasmussen et al., 2017; Zeier & Reiser, 2017). However, the mechanisms linking suPAR to CSIare still being elucidated. Notably, when analyzing the relationship between glycemic control in patients with type 1 diabetes mellitus (T1D) and the risk of developing cardiovascular disease, suPAR has emerged as an important predictor of mortality. Bahrami et al. (2025) evaluated a cohort of individuals with a median age of 50 years and T1D, without known cardiovascular disease, and investigated the association between mortality and circulating levels of suPAR and interleukin-6 (IL-6), assessed at a single baseline time point. The results showed that participants with elevated suPAR or IL-6 had higher mortality, and this risk was even more pronounced when both markers were simultaneously increased, reinforcing their role in mortality and cardiovascular complications in individuals with T1D, even in the absence of diagnosed heart disease. In contrast, (Sowmya et al., 2025), in a cross-sectional study including diabetic individuals and healthy controls, observed no correlation between suPAR levels and glycemic control, suggesting that this marker may not adequately reflect inflammation in younger diabetic adults (25 to 40 years).

Additionally, recent studies have employed proteomic approaches to investigate the association between suPAR levels and the risk of heart failure (HF) in the general population. (Yadalam et al., 2025) followed individuals without previous HF or coronary artery disease for a mean period of 13.7 years. During follow-up, a small proportion of participants developed HF, and the analysis demonstrated that higher suPAR levels were associated with an increased risk of both ischemic and non-ischemic heart failure. Although acute kidney injury (AKI) represents an acute condition, it provides a useful experimental model to investigate suPAR-driven inflammatory signaling pathways. Wang et al. (2025) reported that patients with AKI exhibit significantly elevated suPAR levels. In vitro exposure of HK-2 cells to suPAR induced endoplasmic reticulum stress and increased the expression of apoptosis-associated proteins. These findings were confirmed in vivo, as mice injected intraperitoneally with recombinant suPAR exhibited similar molecular alterations. The authors also observed that FITC-labeled recombinant suPAR colocalizes with the receptor for advanced glycation end products (RAGE) in HK-2 cells, and that RAGE inhibition attenuates suPAR-induced endoplasmic reticulum stress. These results suggest a possible direct interaction between suPAR and RAGE, indicating that this receptor may mediate part of the intracellular signaling triggered by suPAR (Wang et al., 2025) (Fig. 1).

Taken together, these findings indicate that suPAR, through interactions with multiple signaling pathways, contributes to systemic pathological outcomes associated with persistent inflammatory activation. Although not disease-specific, elevated suPAR levels have been reported across a wide range of inflammatory and infectious conditions, reflecting sustained activation of central inflammatory pathways (Rasmussen et al., 2021). uPAR is predominantly expressed on immune cells, including monocytes, macrophages, neutrophils, and activated T lymphocytes, and its expression is upregulated by innate immune stimuli via NF-κB and AP-1 signaling (Thunø et al., 2009). This process enhances plasminogen activator urokinase receptor (PLAUR) transcription, leading to increased uPAR expression and suPAR release into circulation. The uPA–uPAR complex promotes plasmin generation, activating the complement system and amplifying inflammation, while uPAR interactions with vitronectin, integrins, CK-1, and gC1qR support immune cell adhesion, migration, bradykinin production, and cytokine release (Khan et al., 2006; Rosso, 2008; Smith & Marshall, 2010). Additionally, suPAR directly exerts proinflammatory effects through its D2/D3 domains by interacting with FPRL1 on circulating immune cells (Rasmussen et al., 2021; Resnati et al., 2002). Consequently, elevated suPAR levels reflect both the intensity and chronicity of immune activation and may also be associated with myeloid expansion observed in several pathological conditions (Pietras, 2017). In particular, uPAR interactions with integrins such as MAC1 (also known as αMβ2) facilitate monocyte and neutrophil adhesion to the ECM, further promoting directed migration and chemotaxis toward sites of inflammation (Smith & Marshall, 2010) (Fig. 1).

Considering that suPAR reflects the activation of central and broadly conserved inflammatory pathways, it is plausible that it may also be elevated in diseases affecting the CNS. These disorders share inflammatory mechanisms similar to those observed in the periphery, and the neuroinflammation and systemic immune activation frequently associated with CNS pathologies increase the likelihood of elevated circulating and potentially local suPAR levels.

## Emerging Evidence Linking suPAR To CNS Dysfunction

Recent studies have shown that CSI significantly modulates neurovascular function, primarily by altering the permeability and integrity of the BBB (Galea, 2021). Peripheral inflammatory mediators induce endothelial activation, increase the expression of adhesion molecules, promote infiltration of myeloid cells, and amplify the immune response within the brain parenchyma (Galea, 2021; Thomson et al., 2020; Walker et al., 2023). In addition to these mechanisms, hyperfibrinolysis observed in certain pathologies can also contribute to BBB dysfunction, as excessive fibrinolytic activity increases its permeability through a bradykinin-dependent process (Marcos-Contreras et al., 2016). This dynamic communication between the periphery and the CNS leads to BBB impairment, neurovascular uncoupling, and increased tissue vulnerability, phenomena commonly observed in neuroinflammatory and neurodegenerative conditions (Walker et al., 2023). In this context, it is important to note that suPAR is more appropriately interpreted as a biomarker reflecting the intensity of CSI and fibrinolytic activation, since, to date, there are insufficient data in the literature to describe it as a direct mediator of BBB disruption. Accordingly, elevated circulating suPAR levels may indicate a biological milieu that favors endothelial dysfunction, leukocyte trafficking, and BBB impairment.

Although suPAR has been associated with CSI processes, evidence supporting its role as a biomarker of inflammation related to the CNS remains limited, restricting the understanding of its mechanisms and clinical relevance. However, recent studies have sought to elucidate its potential as a biomarker in neurological disorders (De Almeida et al., 2022; Guduguntla et al., 2024; Sajanti et al., 2025; Shell et al., 2025). One of the scenarios in which this gap has been particularly explored is HIV infection, where several studies have shown that HIV-associated neurocognitive disorders largely arise from persistent inflammation within the CNS and chronic immune activation (De Almeida et al., 2022; Gianella et al., 2019). In this context, De Almeida and colleagues (2022) analyzed cerebrospinal fluid (CSF) and blood from individuals living with HIV and observed that elevated CSF suPAR levels, as well as a higher CSF-to-serum suPAR ratio, correlated with more pronounced working memory deficits. This finding suggests that increased monocyte activation in CNS and CSI may impair this cognitive domain.

CSI has also been linked to reductions in executive function (Campbell et al., 2010; Shell et al., 2025). In a longitudinal study in which adults were followed for 8 to 19 years (median of 17 years), increases in circulating suPAR levels were significantly associated with declines in executive function over time. In contrast, traditional inflammatory markers such as IL-6 and CRP did not appear to be related to executive dysfunction. Baseline levels of these markers at study entry did not predict future cognitive changes; rather, it was the progressive increase in suPAR over the years that was associated with subsequent cognitive decline (Shell et al., 2025). Conversely, the prospective observational study by (Guduguntla et al., 2024) did not find an independent association between suPAR or high-sensitivity CRP and dementia or cognitive decline in a median follow-up of only two years. The contrast between the findings of (Shell et al., 2025) and (Guduguntla et al., 2024) suggests that the divergence is primarily related to follow-up duration and the type of inflammatory measurement analyzed. Together, these data indicate that cognitive changes associated with CSI tend to require long periods to manifest and are more accurately detected by the trajectory of inflammatory markers over time rather than by baseline concentrations.

Regarding neurological disorders, an association has also been observed between suPAR levels and migraine with aura (Tesfay et al., 2024; Yılmaz et al., 2017). Individuals with migraine with aura exhibited suPAR levels 6.7% higher than healthy controls; however, patients with migraine in general, including those without aura, did not differ from controls. These findings suggest that elevated suPAR may reflect inflammatory mechanisms specific to migraine with aura rather than migraine as a whole.

Furthermore, suPAR has been investigated as a prognostic biomarker in brain injuries. A recent prospective study evaluated individuals with aneurysmal subarachnoid hemorrhage, ischemic stroke, and traumatic brain injury (Sajanti et al., 2025). Approximately 5.9 days after injury, blood samples were collected to analyze suPAR and other inflammatory markers, and clinical outcomes were assessed at 90 days using the modified Rankin Scale. All injury groups presented elevated suPAR levels compared with healthy controls, and those with unfavorable outcomes exhibited significantly higher concentrations. No significant differences in suPAR levels were observed among the different injury types, indicating that the marker appears to reflect general inflammation following brain injury rather than disease-specific processes. The study also found that suPAR levels were similar in participants with and without infections and did not correlate with CRP, suggesting that increased suPAR in brain injury does not depend on active infection but may be associated with a state of CSI (Sajanti et al., 2025). These findings are consistent with previous studies identifying suPAR as a prognostic biomarker in acute brain injuries (Onatsu et al., 2017; Śmiłowska et al., 2022). All the data are summarized in Table 1.

Interestingly, elevated suPAR levels in the CNS may be associated with the infiltration of monocytes originating from the periphery. However, it is still difficult to determine the relative contribution of peripheral and central sources to the suPAR levels observed in the CNS. Studies have shown that increased suPAR concentrations in the CSF, as well as elevated CSF/serum ratios, are associated with neurological conditions, suggesting that this molecule may not only reflect peripheral immune activation but also involve local production or accumulation in the CNS. Nevertheless, to date, there is no direct evidence confirming suPAR production by resident brain immune cells, leaving this question open and representing an important area for future investigation.

**Table 1:** Summary of clinical studies evaluating SuPAR in CNS-related conditions, showing associations between elevated SuPAR levels in CSF and/or blood and cognitive or neurological outcomes. Evidence is derived from cross-sectional, prospective, and longitudinal studies with varying follow-up periods. Age values refer to participants with the condition of interest (patient groups). SD indicates standard deviation; IQR, interquartile range; aSAH, aneurysmal subarachnoid hemorrhage

Study (Year)	Population/Condition	Age	Design/Follow-up period	Sample	Main findings
Gianella et al., 2019	HIV	Median 48 years (range 27–63 years)	Observational/2005–2015	CSF and blood	Elevated CSF suPAR correlated with worse working memory
De Almeida et al., 2022	HIV	Median 43 years (IQR 35–48)	Cross-sectional/Not applicable	CSF and blood	Higher CSF-to-serum suPAR ratio associated with cognitive deficits
Shell et al., 2025	General adult population	30–54 years at baseline; 40–71 years at follow-up	Longitudinal/Up to ~ 20 years (W1: 2001–2011; W2: 2017–2024)	Blood	Progressive increase in suPAR associated with decline in executive function
Guduguntla et al., 2024	Older adults	70.5 ± 7.0 years (mean ± SD)	Prospective	Blood	No association between suPAR and dementia in a 2-year follow-up
Yılmaz et al., 2017	Migraine	34.84 ± 9.68 years (mean ± SD)	Cross-sectional/Not applicable	Blood	suPAR increased during migraine attacks; 6.7% higher in migraine with aura
Tesfay et al., 2024	Migraine	44.0 ± 12.2 years (mean ± SD)	Cross-sectional/Not applicable	Blood	
Onatsu et al., 2017	Ischemic Stroke	61 ± 11 years (mean ± SD)	Prospective/ 2005–2009	Blood	Higher suPAR levels in patients with unfavorable outcomes
Smilowska et al., 2022	Ischemic Stroke	70.4 ± 7.9 years (mean ± SD)	Prospective/ Days 1–7 post-stroke	Blood	
(Sajanti et al., 2025)	aSAH, ischemic stroke, TBI	58.4 ± 12.7 years (mean ± SD)	Prospective/ 90 days post-injury	Blood	All injury groups showed elevated suPAR; highest in poor outcomes

## Conclusions and Future Perspectives

This study highlights the role of suPAR as a potential biomarker of CSI, surpassing traditional markers such as CRP and IL-6 in certain contexts. Recent evidence also demonstrates its ability to reflect neuroinflammatory processes and alterations in neurovascular function, particularly when associated with immune cell activation and increased permeability of the BBB. Consistently, suPAR has been identified as a possible indicator of cognitive decline and as a useful marker for detecting inflammation associated with disease severity or clinical outcomes in brain injuries.

However, the literature regarding the mechanisms through which suPAR acts within the CNS remains limited. Possible relationships can be inferred from signaling pathways implicated in peripheral inflammation, which may also play relevant roles in the brain. Existing studies display substantial heterogeneity in follow-up duration, methodological design, types of samples analyzed, and characteristics of the populations investigated. In addition, it is important to note that most studies primarily assess total suPAR, without distinguishing or quantifying the specific role of its D2–D3 domains, which limits the understanding of the molecular mechanisms underlying the observed clinical associations. Moreover, although therapeutic strategies aimed at reducing circulating suPAR levels exist, such as the monoclonal antibody WAL0921 in patients with glomerular kidney diseases, their effects on the CNS remain unknown, leaving an important gap to be explored.

Future research is expected to elucidate the cellular and molecular mechanisms of suPAR activity in neurodegenerative and neuroinflammatory conditions, clarifying whether this molecule functions solely as a biomarker or actively participates in inflammatory cascades. In the context of elevated suPAR levels in the CNS, it will be important to determine whether its origin is exclusively peripheral or whether resident immune cells, such as glial cells, significantly contribute to its production. Additionally, consolidating its predictive and prognostic value and understanding how therapeutic interventions influence its levels will be essential. Advancing this body of evidence may ultimately establish suPAR as a relevant tool at the interface between CSI and brain health.

## Data Availability

No datasets were generated or analysed during the current study.

## References

[CR1] Artusa, F., Lamatsch, S., Phan, M. D., Özdirik, B., Berger, H., Egerer, M., et al. (2025). Soluble urokinase plasminogen activator receptor predicts survival and hepatic decompensation in advanced hepatocellular carcinoma. *Liver International*, *45*(6). 10.1111/liv.70121 10.1111/liv.70121 PMC12046945 40317602

[CR2] Bahrami, H. S. Z., Jørgensen, P. G., Hove, J. D., Dixen, U., Rasmussen, L. J. H., Eugen-Olsen, J., et al. (2025). Soluble urokinase plasminogen activator receptor and interleukin‐6 improves prediction of all‐cause mortality and major adverse cardiovascular events in Type 1 diabetes. *Journal of Internal Medicine,**298*(3), 188–199. 10.1111/joim.20108 40619914 10.1111/joim.20108 PMC12374762

[CR3] Blasi, F., & Carmeliet, P. (2002). uPAR: A versatile signalling orchestrator. *Nature Reviews Molecular Cell Biology*, *3*(12), 932–943. 10.1038/nrm977 12461559 10.1038/nrm977

[CR4] Campbell, I. L., Hofer, M. J., & Pagenstecher, A. (2010). Transgenic models for cytokine-induced neurological disease. *Biochimica et Biophysica Acta - Molecular Basis of Disease,**1802*(10), 903–917. 10.1016/j.bbadis.2009.10.004 10.1016/j.bbadis.2009.10.004 PMC2888861 19835956

[CR5] De Almeida, S. M., Rotta, I., Tang, B., Umlauf, A., Vaida, F., Cherner, M., et al. (2022). Higher cerebrospinal fluid soluble urokinase-type plasminogen activator receptor, but not interferon γ-inducible protein 10, correlate with higher working memory deficits. *JAIDS Journal of Acquired Immune Deficiency Syndromes,**90*(1), 106–114. 10.1097/QAI.0000000000002924 35090158 10.1097/QAI.0000000000002924 PMC8986587

[CR6] Desmedt, S., Desmedt, V., Delanghe, J. R., Speeckaert, R., & Speeckaert, M. M. (2017). The intriguing role of soluble urokinase receptor in inflammatory diseases. *Critical Reviews in Clinical Laboratory Sciences*, *54*(2), 117–133. 10.1080/10408363.2016.1269310 28084848 10.1080/10408363.2016.1269310

[CR7] Furman, D., Campisi, J., Verdin, E., Carrera-Bastos, P., Targ, S., Franceschi, C., et al. (2019). Chronic inflammation in the etiology of disease across the life span. *Nature Medicine,**25*(12), 1822–1832. 10.1038/s41591-019-0675-0 31806905 10.1038/s41591-019-0675-0 PMC7147972

[CR8] Galea, I. (2021). The blood–brain barrier in systemic infection and inflammation. *Cellular & Molecular Immunology*, *18*(11), 2489–2501. 10.1038/s41423-021-00757-x 34594000 10.1038/s41423-021-00757-x PMC8481764

[CR9] Gianella, S., Letendre, S. L., Iudicello, J., Franklin, D., Gaufin, T., Zhang, Y., et al. (2019). Plasma (1 → 3)-β-d-glucan and suPAR levels correlate with neurocognitive performance in people living with HIV on antiretroviral therapy: A CHARTER analysis. *Journal of NeuroVirology,**25*(6), 837–843. 10.1007/s13365-019-00775-6 31297727 10.1007/s13365-019-00775-6 PMC6923595

[CR10] Guduguntla, B. A., Vasbinder, A., Anderson, E., Azam, T. U., Blakely, P., Webster, N. J., et al. (2024). Biomarkers of chronic inflammation and cognitive decline: A prospective observational study. *Alzheimer’s & Dementia: Diagnosis, Assessment & Disease Monitoring*. 10.1002/dad2.12568 10.1002/dad2.12568 PMC10964918 38532827

[CR11] Haupt, T. H., Kallemose, T., Ladelund, S., Rasmussen, L. J. H., Thorball, C. W., Andersen, O., et al. (2014). Risk factors associated with serum levels of the inflammatory biomarker soluble urokinase plasminogen activator receptor in a general population. *Biomarker Insights,**9*, Article BMI.S19876. 10.4137/BMI.S19876 10.4137/BMI.S19876 PMC4269129 25574132

[CR12] Hayek, S. S., Leaf, D. E., Samman Tahhan, A., Raad, M., Sharma, S., Waikar, S. S., et al. (2020). Soluble urokinase receptor and acute kidney injury. *The New England Journal of Medicine,**382*(5), 416–426. 10.1056/NEJMoa1911481 31995687 10.1056/NEJMoa1911481 PMC7065830

[CR13] Hodges, G. W., Bang, C. N., Wachtell, K., Eugen-Olsen, J., & Jeppesen, J. L. (2015). suPAR: A new biomarker for cardiovascular disease? *Canadian Journal of Cardiology,**31*(10), 1293–1302. 10.1016/j.cjca.2015.03.023 26118447 10.1016/j.cjca.2015.03.023

[CR14] Hynes, R. O. (2009). The extracellular matrix: Not just pretty fibrils. *Science,**326*(5957), 1216–1219. 10.1126/science.1176009 19965464 10.1126/science.1176009 PMC3536535

[CR15] Jo, M., Takimoto, S., Montel, V., & Gonias, S. L. (2009). The urokinase receptor promotes cancer metastasis independently of urokinase-type plasminogen activator in mice. *The American Journal of Pathology,**175*(1), 190–200. 10.2353/ajpath.2009.081053 19497996 10.2353/ajpath.2009.081053 PMC2708805

[CR16] Khan, M. M., Bradford, H. N., Isordia-Salas, I., Liu, Y., Wu, Y., Espinola, R. G., et al. (2006). Mac-1, and gC1qR in Monocytes. *Arteriosclerosis Thrombosis and Vascular Biology*, *26*(10), 2260–2266. 10.1161/01.ATV.0000240290.70852.c0 10.1161/01.ATV.0000240290.70852.c0 PMC2637648 16902163

[CR17] Kular, J. K., Basu, S., & Sharma, R. I. (2014). The extracellular matrix: Structure, composition, age-related differences, tools for analysis and applications for tissue engineering. *Journal of Tissue Engineering*. 10.1177/2041731414557112 25610589 10.1177/2041731414557112 PMC4883592

[CR18] Marcos-Contreras, O. A., de Martinez Lizarrondo, S., Bardou, I., Orset, C., Pruvost, M., Anfray, A., et al. (2016). Hyperfibrinolysis increases blood–brain barrier permeability by a plasmin- and bradykinin-dependent mechanism. *Blood,**128*(20), 2423–2434. 10.1182/blood-2016-03-705384 27531677 10.1182/blood-2016-03-705384

[CR19] Mehta, A., Desai, S. R., Ko, Y., Liu, C., Dhindsa, D. S., Nayak, A., et al. (2020). Sex differences in Circulating soluble Urokinase-Type plasminogen activator receptor (suPAR) levels and adverse outcomes in coronary artery disease. *Journal of the American Heart Association*, *9*(5). 10.1161/JAHA.119.015457 10.1161/JAHA.119.015457 PMC7335555 32089048

[CR20] Onatsu, J., Taina, M., Mustonen, P., Hedman, M., Muuronen, A., Arponen, O., et al. (2017). Soluble urokinase-type plasminogen activator receptor predicts all-cause 5-year mortality in ischemic stroke and TIA. *In Vivo,**31*(3), 381–386. 10.21873/invivo.11070 28438866 10.21873/invivo.11070 PMC5461448

[CR21] Pietras, E. M. (2017). Inflammation: A key regulator of hematopoietic stem cell fate in health and disease. *Blood,**130*(15), 1693–1698. 10.1182/blood-2017-06-780882 28874349 10.1182/blood-2017-06-780882 PMC5639485

[CR22] Ploug, M., & Ellis, V. (1994). Structure—function relationships in the receptor for urokinase-type plasminogen activator comparison to other members of the Ly‐6 family and snake venom α‐neurotoxins. *FEBS Letters*, *349*(2), 163–168. 10.1016/0014-5793(94)00674-1 8050560 10.1016/0014-5793(94)00674-1

[CR23] Rasmussen, L. J. H., Caspi, A., Ambler, A., Danese, A., Elliott, M., Eugen-Olsen, J., et al. (2021). Association between elevated suPAR, a new biomarker of inflammation, and accelerated aging. *The Journals of Gerontology: Series A,**76*(2), 318–327. 10.1093/gerona/glaa178 10.1093/gerona/glaa178 PMC7812430 32766674

[CR24] Rasmussen, L. J. H., Schultz, M., Gaardsting, A., Ladelund, S., Garred, P., Iversen, K., et al. (2017). Inflammatory biomarkers and cancer: CRP and suPAR as markers of incident cancer in patients with serious nonspecific symptoms and signs of cancer. *International Journal of Cancer,**141*(1), 191–199. 10.1002/ijc.30732 28393357 10.1002/ijc.30732 PMC5518177

[CR25] Reiser, J., Hayek, S. S., & Sever, S. (2026). The role of SuPAR and related proteins in kidney, heart diseases, and diabetes. *Journal of Clinical Investigation*, *136*(1). 10.1172/JCI197141 10.1172/JCI197141 PMC12721894 41480757

[CR26] Resnati, M., Pallavicini, I., Wang, J. M., Oppenheim, J., Serhan, C. N., Romano, M., & Blasi, F. (2002). The fibrinolytic receptor for urokinase activates the G protein-coupled chemotactic receptor FPRL1/LXA4R. *Proceedings of the National Academy of Sciences,**99*(3), 1359–1364. 10.1073/pnas.022652999 10.1073/pnas.022652999 PMC122195 11818541

[CR27] Rosso, M. D. (2008). The plasminogen activation system in inflammation. *Frontiers in Bioscience,**Volume*(13), 4667. 10.2741/3032 10.2741/3032 18508538

[CR28] Sajanti, A., Hellström, S., Bennett, C., et al. (2025). Soluble urokinase-type plasminogen activator receptor and inflammatory biomarker response with prognostic significance after acute neuronal injury – A prospective cohort study. *Inflammation,**48*(4), 2217–2229. 10.1007/s10753-024-02185-1 39540961 10.1007/s10753-024-02185-1 PMC12336084

[CR29] Saraswathibhatla, A., Indana, D., & Chaudhuri, O. (2023). Cell–extracellular matrix mechanotransduction in 3D. *Nature Reviews Molecular Cell Biology*, *24*(7), 495–516. 10.1038/s41580-023-00583-1 36849594 10.1038/s41580-023-00583-1 PMC10656994

[CR30] Sehgal, T., Anand, A., Vijayabupathy, G., & Khan, M. (2024). Soluble urokinase plasminogen activator receptor: A useful marker for predicting mortality in COVID-19 patients. *Cureus*. 10.7759/cureus.74438 39723295 10.7759/cureus.74438 PMC11669473

[CR31] Shell, A., Vize, C., Gianaros, P., Rasmussen, L. J. H., & Marsland, A. L. (2025). Executive function and soluble urokinase-type plasminogen activator receptor (suPAR): A longitudinal study of midlife adults. *Brain, Behavior, and Immunity,**129*, 537–546. 10.1016/j.bbi.2025.06.030 40578537 10.1016/j.bbi.2025.06.030 PMC12977348

[CR32] Śmiłowska, K., Śmiłowski, M., Partyka, R., Kokocińska, D., & Jałowiecki, P. (2022). Personalised approach to diagnosing and managing ischemic stroke with a plasma-soluble urokinase-type plasminogen activator receptor. *Journal of Personalized Medicine,**12*(3), Article 457. 10.3390/jpm12030457 35330458 10.3390/jpm12030457 PMC8953259

[CR33] Smith, H. W., & Marshall, C. J. (2010). Regulation of cell signalling by uPAR. *Nature Reviews Molecular Cell Biology,**11*(1), 23–36. 10.1038/nrm2821 20027185 10.1038/nrm2821

[CR34] Sowmya, A., Reshma, K., Sudha, K., & Himaniv, K. (2025). Correlation of soluble urokinase plasminogen activator receptor (suPAR) with HbA1c in predicting vascular complications in young adults - A cross sectional study. *La Clinica Terapeutica*, *176*(5), 562–566. 10.7417/CT.2025.5266 40996003 10.7417/CT.2025.5266

[CR35] Tesfay, B., Ashina, H., Christensen, R. H., Al-Khazali, H. M., Karlsson, W. K., Amin, F. M., et al. (2024). Association of plasma soluble urokinase plasminogen activator receptor concentrations and migraine with aura: A REFORM study. *Brain Communications*. 10.1093/braincomms/fcae475 10.1093/braincomms/fcae475 PMC11831075 39963289

[CR36] Thomson, C. A., McColl, A., Graham, G. J., & Cavanagh, J. (2020). Sustained exposure to systemic endotoxin triggers chemokine induction in the brain followed by a rapid influx of leukocytes. *Journal of Neuroinflammation,**17*(1), Article 94. 10.1186/s12974-020-01759-8 32213184 10.1186/s12974-020-01759-8 PMC7098135

[CR37] Thunø, M., Macho, B., & Eugen-Olsen, J. (2009). SuPAR: The molecular crystal ball. *Disease Markers*, *27*(3), 157–172. 10.3233/DMA-2009-0657 19893210 10.3233/DMA-2009-0657 PMC3835059

[CR38] Walker, K. A., Le Page, L. M., Terrando, N., Duggan, M. R., Heneka, M. T., & Bettcher, B. M. (2023). The role of peripheral inflammatory insults in Alzheimer’s disease: A review and research roadmap. *Molecular Neurodegeneration,**18*(1), Article 37. 10.1186/s13024-023-00627-2 37277738 10.1186/s13024-023-00627-2 PMC10240487

[CR39] Wang, B., Wang, J., Qi, C., Gao, C., Wang, Y., Zan, Y., et al. (2025). Soluble urokinase plasminogen activator receptor promotes endoplasmic reticulum stress and apoptosis susceptibility through RAGE in sepsis acute kidney injury. *Molecular Medicine,**31*(1), Article 296. 10.1186/s10020-025-01352-w 41013175 10.1186/s10020-025-01352-w PMC12465643

[CR40] Yadalam, A. K., Gold, M. E., Patel, K. J., Liu, C., Razavi, A. C., Jain, V., et al. (2025). Proteomics-based soluble urokinase plasminogen activator receptor levels are associated with incident heart failure risk. *JACC: Advances,**4*(1), Article 101442. 10.1016/j.jacadv.2024.101442 39737138 10.1016/j.jacadv.2024.101442 PMC11683231

[CR41] Ye, S. (2001). Serpins and other covalent protease inhibitors. *Current Opinion in Structural Biology*, *11*(6), 740–745. 10.1016/S0959-440X(01)00275-5 11751056 10.1016/s0959-440x(01)00275-5

[CR42] Yılmaz, N., Yılmaz, M., Sirin, B., Yılmaztekin, S., & Kutlu, G. (2017). The relationship between levels of plasma-soluble urokinase plasminogen activator receptor (suPAR) and presence of migraine attack and aura. *Journal of Receptors and Signal Transduction*, *37*(5), 447–452. 10.1080/10799893.2017.1328440 28553881 10.1080/10799893.2017.1328440

[CR43] Zeier, M., & Reiser, J. (2017). SuPAR and chronic kidney disease—a podocyte story. *Pflügers Archiv - European Journal of Physiology*, *469*(7–8), 1017–1020. 10.1007/s00424-017-2026-7 28689240 10.1007/s00424-017-2026-7

[CR44] Zotova, N., Zhuravleva, Y., Chereshnev, V., & Gusev, E. (2023). Acute and chronic systemic inflammation: Features and differences in the pathogenesis, and integral criteria for verification and differentiation. *International Journal of Molecular Sciences,**24*(2), Article 1144. 10.3390/ijms24021144 36674657 10.3390/ijms24021144 PMC9862412

